# Regression of Tricuspid Regurgitation after Pulmonary Endarterectomy

**DOI:** 10.1186/1749-8090-10-S1-A91

**Published:** 2015-12-16

**Authors:** Jaroslav Lindner, David Ambrož, Matúš Nižňanský, Tomáš Paleček, Tomáš Prskavec, Onřej Pecha, Pavel Jansa

**Affiliations:** 1 2nd Surgical Department - Department of Cardiovascular Surgery, 1st Faculty of Medicine Charles University in Prague, CZ128 00, Czech Republic; 2 2nd Department of Internal Medicine, Department of Cardiology and Angiology, 1st Faculty of Medicine Charles University in Prague, CZ128 00, Czech Republic; 3 Technology Centre of the Academy of Sciences, Prague, CZ 117 20, Czech Republic

## Background/Introduction

Pulmonary endarterectomy (PEA) is the effective treatment for chronic thromboembolic pulmonary hypertension (CTEPH). Preoperative echocardiographic images often reveal severe tricuspid regurgitation, which regresses after successful surgical procedure. This is the main reason why tricuspid valve surgery is not performed along with PEA.

## Aims/Objectives

We focused on the development of tricuspid regurgitation. Our aim was to analyze our strategy of treatment that does not involve tricuspid valve surgery.

## Method

We performed analysis of patients who were operated on during the period between years 2004-2011. The data was collected from a set of 100 patients, 65 men and 35 women. Only 1 patient underwent concomitant tricuspid valve surgery - tricuspid valve repair. We analyzed different echocardiographic parameters of all our patients prior to operation and then 1 month, and 3 years after the procedure. We were mostly interested into the degree of tricuspid regurgitation, then the right ventricle function and presence of pulmonary hypertension. Repeated measures ANOVA with Fisher post-hoc test was used for evaluating the differences between the consecutive measurements.

## Results

Average tricuspid regurgitation was 2.42 preoperatively, then significantly decreased to 1.44 postoperatively (p < 0.001) and negligibly increased to 1.45 three years after the procedure (p = 0.893). Almost 60% of patients had preoperatively severe tricuspid regurgitation (at least 2.5). After the operation 15% of patients had severe tricuspid regurgitation and after three years it was only 12%. Patients with severe regurgitation (at least 2.5) had in average PASP of 67.4 mmHg, patients with regurgitation greater than 3.0 had PASP over 68,4 mmHg. Mean FAC parameter was significantly elevated from 28.05 preoperatively to 34.87 postoperatively (p < 0.001) and slightly increased to 38.47 (p = 0.350) three years after the procedure.

## Discussion/Conclusion

The results of our analysis confirm that our strategy not to perform any tricuspid valve surgery unless there is an organic cause of the valve disease was correct. The functional tricuspid insufficiency, even if severe, regresses after successful PEA and pulmonary artery pressure reduction. None of our patients was indicated to undergo tricuspid valve surgery as a redo procedure following PEA.

